# K_0.53_Mn_2.37_Fe_1.24_(PO_4_)_3_

**DOI:** 10.1107/S1600536810051238

**Published:** 2010-12-11

**Authors:** Mourad Hidouri, Mongi Ben Amara

**Affiliations:** a Faculté des Sciences de Monastir, 5019 Monastir, Tunisia

## Abstract

During an attempt to crystallize potassium manganese diiron phosphate KMnFe_2_(PO_4_)_3_ by the flux method, a new phase, potassium dimanganese iron triphosphate, K_0.53_Mn_2.37_Fe_1.24_(PO_4_)_3_, was isolated. This phase, whose composition was confirmed by ICP analysis, is isotypic with the alluaudite-like phosphates, thus it exhibits the (*A*2)(*A*′2)(*A*1)(*A*′1)(*A*′′1)(*M*1)(*M*2)_2_(PO_4_)_3_ general formula. The site occupancies led to the following cation distribution: 0.53 K on *A*′2 (site symmetry 2), 0.31 Mn on *A*′′1, 1.0 Mn on *M*1 (site symmetry 2) and (0.62 Fe + 0.38 Mn) on *M*2. The structure is built up from infinite chains of edge-sharing *M*1O_6_ and *M*2O_6_ octa­hedra. These chains run along [10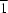] and are connected by two different PO_4_ tetrahedra, one of which exhibits 2 symmetry. The resulting three-dimensional framework delimits large tunnels parallel to [001], which are partially occupied by the K^+^ and Mn^2+^ cations.

## Related literature

For the alluaudite structure, see: Fisher (1955 ▶); Moore (1971 ▶); Chouaibi *et al.* (2001 ▶); Corbin *et al.* (1986 ▶); Lee & Ye (1997 ▶); Hidouri *et al.* (2003 ▶, 2004 ▶, 2008 ▶) Antenucci *et al.* (1993 ▶, 1995 ▶); For P—O distances, see: Baur (1974 ▶). For bond-valence sums, see: Brown & Altermatt (1985 ▶). For ionic radii, see: Shannon (1976 ▶).

## Experimental

### 

#### Crystal data

K_0.53_Mn_2.37_Fe_1.24_(PO_4_)_3_
*M* *_r_* = 505.28
Monoclinic, 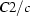

*a* = 12.272 (2) Å
*b* = 12.606 (2) Å
*c* = 6.416 (4) Å
β = 114.87 (2)°
*V* = 900.5 (6) Å^3^
*Z* = 4
Mo *K*α radiation
μ = 6.07 mm^−1^
*T* = 293 K
0.43 × 0.09 × 0.02 mm

#### Data collection

Enraf–Nonius TurboCAD-4 diffractometer
Absorption correction: refined from Δ*F* (Parkin *et al.*, 1995 ▶) *T*_min_ = 0.42, *T*_max_ = 0.81
1754 measured reflections
1308 independent reflections
1047 reflections with *I* > 2σ(*I*)
*R*_int_ = 0.042
2 standard reflections every 120 min  intensity decay: 1%

#### Refinement

*R*[*F* ^2^ > 2σ(*F* ^2^)] = 0.037
*wR*(*F* ^2^) = 0.089
*S* = 1.07
1308 reflections
102 parameters
2 restraints
Δρ_max_ = 0.78 e Å^−3^
Δρ_min_ = −0.68 e Å^−3^

### 

Data collection: *CAD-4 EXPRESS* (Enraf–Nonius, 1994 ▶); cell refinement: *CAD-4 EXPRESS*; data reduction: *XCAD4* (Harms & Wocadlo, 1995 ▶); program(s) used to solve structure: *SIR92* (Altomare *et al.*, 1993 ▶); program(s) used to refine structure: *SHELXL97* (Sheldrick, 2008 ▶); molecular graphics: *SHELXTL* (Sheldrick, 2008 ▶); software used to prepare material for publication: *SHELXL97*.

## Supplementary Material

Crystal structure: contains datablocks I, global. DOI: 10.1107/S1600536810051238/br2150sup1.cif

Structure factors: contains datablocks I. DOI: 10.1107/S1600536810051238/br2150Isup2.hkl

Additional supplementary materials:  crystallographic information; 3D view; checkCIF report

## Figures and Tables

**Fig. 1.**
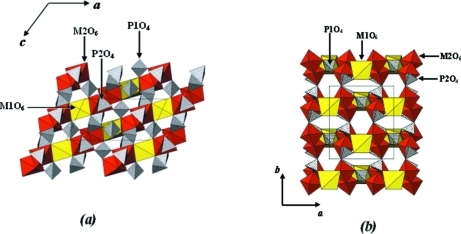
Polyhedral representations of the K0.53Mn2.37Fe1.24(PO4)3 structure as projected along [010] (a) and along [001] (b). M1O6, M2O6 are represented by yellow and red octahedra,respectively and PO4 by crossed tetrahedra.

**Fig. 2.**
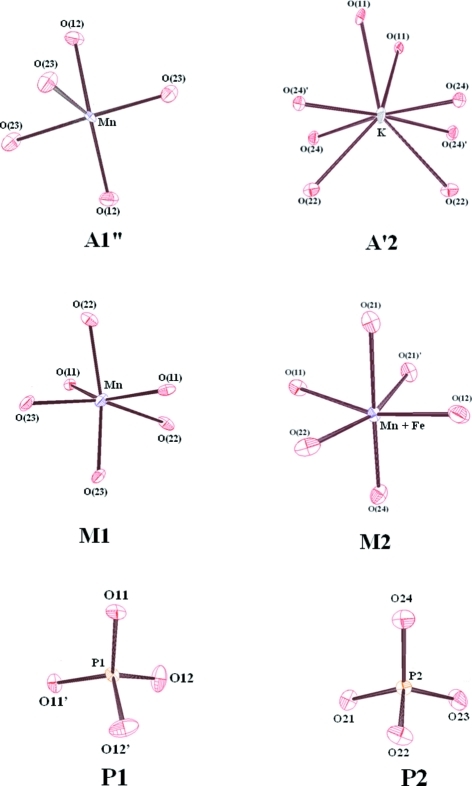
The environments of the A'1, A"2, M1, M2, P1 and P2 sites showing the anisotropic atomic displacements. The thermal ellipsoids are drown at 50 % probability level.

## supplementary crystallographic information

### Comment

The term alluaudite is referred to both natural and synthetic phosphates of compositions (Na^+^)(Na^+^, Ca^2+^)(*M*^2+^)(Fe^2+^,Fe^3+^)_2_(PO_4_)_3_ where *M*^2+^ is a divalent cation. The first detailed structural description was reported in 1971 by Moore (Moore, 1971) who proposed the structural formula (X2)(X1)(*M*1)(*M*2)_2_(PO_4_)_3_. X1 and X2 are cationic sites available to large monovalent and divalent cations such as Na^+^ and Ca^2+^ while *M*1 and *M*2 are octahedral sites containing a distribution of divalent and trivalent cations of moderate size such as Mn^2+^, Fe^2+^ or Fe^3+^. More recently, detailed structural analysis of several alluaudites demonstrated that the X2 site has two distinct positions labeled *A*2 (0, 0, 0) and *A*'2 (0,~0, 1/4) in a tunnel at (0, 0, *z*) and X1 has three distinct positions, labeled *A*1 (1/2, 0, 0), *A*'1 (0,~1/2, 1/4) and *A*''1 (*x*, *y*, *z*) in a tunnel at (1/2, 0, *z*). The general formula of Moore was then reformulated as [(*A*2)(*A*'2)][(*A*1)(*A*'1)(*A*''1)](*M*1)(*M*2)_2_(PO_4_)_3_. The crystal structure consists of *M*2_2_O_10_ bioctahedral units of edge-sharing *M*2O_6_ octahedra, sharing opposite edges with *M*1O_6_ octahedra that form zigzag chains of a sequence –*M*(2)—*M*(2)—*M*(1)-, running along the [1 0 - 1] direction. Adjacent chains are linked by the phosphate tetrahedra leading to what have been described as "pleated sheets" perpendicular to the [0 1 0] direction (Fig. 1(b)). These sheets are connected by the phosphate groups giving rise to a three-dimensional framework with two sets of tunnels parallel to [001] (Fig. 1(b)).

The title phase K_0.53_Mn_2.37_Fe_1.24_(PO_4_)_3_ was isolated during an attempt to synthesize KMnFe_2_(PO_4_)_3_ and its structure has shown to be of the alluaudite type. The site occupancy factors indicated to the following cation distribution: 0.53 K on *A*'2, 0.31 Mn on *A*''1, 1.0 Mn on *M*1 and (0.62 Fe + 0.38 Mn) on *M*2. The partial occupancy of the large A sites has already been observed in several alluaudites being attributed to the great flexibility of these sites which allows them to be filled totally, partially or left vacant without significant influence on the alluaudite framework. Assuming a maximum gap in the cation-oxygen distances, the envronment of the *A*'2 site (figure 2) consists of eight oxygen atoms forming what has been called by Moore as a gable desphenoid (Moore, 1971). That of the *A*''1 site (figure 2) onsists of five O atoms forming a distorted trigonal bipyramid. The fivefold coordination of this site which is, to the best of our knowledge, observed for the first time in an alluaudite-like compound can be attributed to the small size of the Mn^2+^ cation. Both the *M*1 and *M*2 sites are octahedrally coordinated (figure 2). From the *M*1—O distances and *cis* O—*M*1—O angles, one can deduce that the *M*1O_6_ octahedron is strongly distorted. However, the mean *M*1—O> mean distance of 2.238 Å is close to that 2.23 Å predicted by Shannon for octahedral Mn^2+^ cations (Shannon, 1976). The *M*2—O distances and *cis* O—*M*2—O angles show the *M*2O_6_ octahedron to be less distorted than *M*1O_6_. The <*M*(2)—O> mean distance (2.068 Å) is between 2.03 Å and 2.23 Å, calculated by Shannon (Shannon, 1976) for the Fe^3+^ and Mn^2+^ cations, respectively. This result confirms the presence of both atoms on the *M*(2) site. The PO_4_ tetrahedra have classical P—O distances with an overall value of 1.537 Å close to that 1.537 Å, assigned by Baur for the monophosphate groups (Baur, 1974). The Bond Valence Sums (BVS) were calculated for all cationic sites by the Brown and Altermatt method (Brown *et al.*, 1985). The analysis of the sums for the *M*2 site, around Fe^3+^ and Mn^2+^ led to valence sums of 2.87 and 2.64, respectively which corresponds to occupation numbers of 0.71 and 0.29, very close to the x-ray values of 0.62 and 0.38. The sum around Mn3 is 1.34 and around K is 0.42. These are poor because of the partial occupancy but unfortunately these values cannot be used to estimate the occupancy. The sums around P1 and P2 of 4.92 and 4.98, respectively around the O sites (from 1.91 to 2.12) are consistent with the predicted ones of 5 for P and 2 for O. In summery, the valence calculation results gave a good confirmation of the structure, including the assigned oxidation states which cannot be determined by x-ray analysis.

### Experimental

Single crystals of the title phase were extracted from a mixture of nominal composition KMnFe_2_(PO_4_)_3_. The latter was prepared by the flux method starting from a mixture of 2.042 g of KNO_3_, 2.589 g of Mn(NO_3_)_2_.6H_2_O, 8.245 g of Fe(NO_3_)_3_.9H_2_O, 4.002 g of (NH_4_)_2_HPO_4_ and 0.719 g of MoO_3_. These reactants were dissolved in nitric acid and the solution obtained was dried for 24 h at 353 K. The obtained dry residue was ground in an agate mortar to ensure its best homogeneity, then heated in a platinum crucible to 673 K for 24 h in order to remove the decomposition products: NH_3_ and H_2_O. The sample was then reground, melted at 1173 K for 1 h and subsequently cooled at a 10 °.h^-1^ rate to 673 K. The final product was washed with warm water in order to dissolve the flux. From the mixture, dark brown and hexagonally shaped crystals were extracted. Their analysis using ICP confirmed the presence of only K, Mn, Fe and P in atomic ratio of 0.53:2.37:1.24:3, in accordance with the K_0.53_Mn_2.37_Fe_1.24_(PO_4_)_3_ composition.

### Crystal data

**Table:**

K_0.53_Mn_2.37_Fe_1.24_(PO_4_)_3_	*F*(000) = 957
*M**_r_* = 505.28	*D*_x_ = 3.727 Mg m^−^^3^
Monoclinic, *C*2/*c*	Mo *K*α radiation, λ = 0.71073 Å
Hall symbol: -C 2yc	Cell parameters from 25 reflections
*a* = 12.272 (2) Å	θ = 9.8–14.4°
*b* = 12.606 (2) Å	µ = 6.07 mm^−^^1^
*c* = 6.416 (4) Å	*T* = 293 K
β = 114.87 (2)°	Hexagonal, brown
*V* = 900.5 (6) Å^3^	0.43 × 0.09 × 0.02 mm
*Z* = 4	

### Data collection

**Table:**

Enraf–Nonius TurboCAD-4 diffractometer	1047 reflections with *I* > 2σ(*I*)
Radiation source: fine-focus sealed tube	*R*_int_ = 0.042
graphite	θ_max_ = 30.0°, θ_min_ = 2.4°
non–profiled ω/2θ scans	*h* = −17→15
Absorption correction: part of the refinement model (Δ*F*) (Parkin *et al.*, 1995)	*k* = 0→17
*T*_min_ = 0.42, *T*_max_ = 0.81	*l* = 0→9
1754 measured reflections	2 standard reflections every 120 min
1308 independent reflections	intensity decay: 1%

### Refinement

**Table:**

Refinement on *F*^2^	Primary atom site location: structure-invariant direct methods
Least-squares matrix: full	Secondary atom site location: difference Fourier map
*R*[*F*^2^ > 2σ(*F*^2^)] = 0.037	*w* = 1/[σ^2^(*F*_o_^2^) + (0.0451*P*)^2^] where *P* = (*F*_o_^2^ + 2*F*_c_^2^)/3
*wR*(*F*^2^) = 0.089	(Δ/σ)_max_ = 0.001
*S* = 1.07	Δρ_max_ = 0.78 e Å^−^^3^
1308 reflections	Δρ_min_ = −0.68 e Å^−^^3^
102 parameters	Extinction correction: *SHELXL97* (Sheldrick, 2008)
2 restraints	Extinction coefficient: 0.0009 (4)

### Special details

**Table:**

Geometry. All e.s.d.'s (except the e.s.d. in the dihedral angle between two l.s. planes) are estimated using the full covariance matrix. The cell e.s.d.'s are taken into account individually in the estimation of e.s.d.'s in distances, angles and torsion angles; correlations between e.s.d.'s in cell parameters are only used when they are defined by crystal symmetry. An approximate (isotropic) treatment of cell e.s.d.'s is used for estimating e.s.d.'s involving l.s. planes.
Refinement. Refinement of *F*^2^ against ALL reflections. The weighted *R*-factor *wR* and goodness of fit *S* are based on *F*^2^, conventional *R*-factors *R* are based on *F*, with *F* set to zero for negative *F*^2^. The threshold expression of *F*^2^ > σ(*F*^2^) is used only for calculating *R*-factors(gt) *etc*. and is not relevant to the choice of reflections for refinement. *R*-factors based on *F*^2^ are statistically about twice as large as those based on *F*, and *R*- factors based on ALL data will be even larger.

### Fractional atomic coordinates and isotropic or equivalent isotropic displacement parameters (Å^2^)

**Table:**

	*x*	*y*	*z*	*U*_iso_*/*U*_eq_	Occ. (<1)
K	0.0000	−0.0116 (2)	0.2500	0.0229 (7)	0.531 (5)
Mn1	0.0000	0.26376 (7)	0.2500	0.0156 (2)	
Fe2	0.22489 (4)	0.15521 (4)	0.13977 (8)	0.01004 (16)	0.6217 (6)
Mn2	0.22489 (4)	0.15521 (4)	0.13977 (8)	0.01004 (16)	0.3783 (7)
Mn3	−0.02360 (17)	0.49774 (17)	0.0393 (4)	0.0209 (5)	0.3064 (12)
P1	0.0000	0.28642 (11)	−0.2500	0.0096 (3)	
O11	0.0479 (2)	0.2160 (2)	−0.0331 (4)	0.0138 (5)	
O12	−0.0942 (3)	0.3615 (2)	−0.2314 (6)	0.0251 (7)	
P2	0.24436 (8)	0.10810 (7)	0.63905 (15)	0.0093 (2)	
O21	0.2272 (2)	0.1782 (2)	0.8220 (4)	0.0156 (6)	
O22	0.1735 (3)	0.1629 (2)	0.4048 (4)	0.0169 (6)	
O23	0.3783 (2)	0.1019 (2)	0.6976 (6)	0.0255 (7)	
O24	0.1930 (2)	−0.0019 (2)	0.6349 (5)	0.0178 (6)	

### Atomic displacement parameters (Å^2^)

**Table:**

	*U*^11^	*U*^22^	*U*^33^	*U*^12^	*U*^13^	*U*^23^
K	0.0093 (10)	0.0218 (13)	0.0260 (13)	0.000	−0.0040 (9)	0.000
Mn1	0.0115 (4)	0.0202 (4)	0.0154 (4)	0.000	0.0058 (3)	0.000
Fe2	0.0065 (2)	0.0138 (3)	0.0065 (3)	0.00016 (18)	−0.00043 (19)	0.0008 (2)
Mn2	0.0065 (2)	0.0138 (3)	0.0065 (3)	0.00016 (18)	−0.00043 (19)	0.0008 (2)
Mn3	0.0156 (12)	0.0164 (9)	0.0165 (11)	−0.0002 (9)	−0.0070 (7)	0.0014 (9)
P1	0.0060 (5)	0.0140 (6)	0.0038 (5)	0.000	−0.0028 (4)	0.000
O11	0.0087 (11)	0.0193 (13)	0.0066 (11)	−0.0027 (10)	−0.0033 (9)	0.0028 (10)
O12	0.0149 (13)	0.0245 (15)	0.0301 (17)	0.0025 (12)	0.0037 (13)	−0.0119 (14)
P2	0.0065 (4)	0.0123 (4)	0.0050 (4)	0.0004 (3)	−0.0016 (3)	0.0012 (3)
O21	0.0128 (12)	0.0235 (14)	0.0078 (11)	−0.0030 (11)	0.0017 (10)	−0.0038 (11)
O22	0.0244 (14)	0.0141 (14)	0.0048 (11)	0.0003 (11)	−0.0010 (10)	−0.0007 (10)
O23	0.0114 (13)	0.0189 (15)	0.047 (2)	0.0003 (11)	0.0132 (14)	−0.0018 (15)
O24	0.0127 (12)	0.0184 (14)	0.0171 (13)	−0.0013 (11)	0.0012 (11)	0.0030 (11)

### Geometric parameters (Å, °)

**Table:**

K—O24	2.608 (3)	Mn1—O22^i^	2.315 (3)
K—O24^i^	2.608 (3)	Mn1—O22	2.315 (3)
K—O24^ii^	2.767 (3)	Fe2—O24^ii^	1.970 (3)
K—O24^iii^	2.767 (3)	Fe2—O12^x^	2.027 (3)
K—O11^iv^	2.869 (4)	Fe2—O22	2.049 (3)
K—O11^v^	2.869 (4)	Fe2—O21^xi^	2.071 (3)
K—O22	2.929 (3)	Fe2—O11	2.123 (3)
K—O22^i^	2.929 (3)	Fe2—O21^vi^	2.167 (3)
Mn3—O23^vi^	2.253 (4)	P1—O12^xii^	1.537 (3)
Mn3—O23^vii^	2.256 (4)	P1—O12	1.537 (3)
Mn3—O12^viii^	2.294 (4)	P1—O11^xii^	1.544 (3)
Mn3—O12	2.335 (4)	P1—O11	1.544 (3)
Mn3—O23^ix^	2.400 (4)	P2—O24	1.519 (3)
Mn1—O23^vi^	2.189 (3)	P2—O23	1.526 (3)
Mn1—O23^vii^	2.189 (3)	P2—O22	1.547 (3)
Mn1—O11	2.215 (3)	P2—O21	1.553 (3)
Mn1—O11^i^	2.215 (3)		
			
O24—K—O24^i^	174.63 (16)	O23^vi^—Mn1—O11	86.39 (11)
O24—K—O24^ii^	73.24 (8)	O23^vii^—Mn1—O11	118.95 (12)
O24^i^—K—O24^ii^	106.42 (8)	O23^vi^—Mn1—O11^i^	118.95 (12)
O24—K—O24^iii^	106.42 (8)	O23^vii^—Mn1—O11^i^	86.39 (11)
O24^i^—K—O24^iii^	73.24 (8)	O11—Mn1—O11^i^	148.43 (15)
O24^ii^—K—O24^iii^	172.95 (16)	O23^vi^—Mn1—O22^i^	159.42 (10)
O24—K—O11^iv^	114.87 (10)	O23^vii^—Mn1—O22^i^	85.06 (10)
O24^i^—K—O11^iv^	70.34 (8)	O11—Mn1—O22^i^	90.70 (10)
O24^ii^—K—O11^iv^	87.10 (9)	O11^i^—Mn1—O22^i^	71.87 (10)
O24^iii^—K—O11^iv^	99.26 (10)	O23^vi^—Mn1—O22	85.06 (10)
O24—K—O11^v^	70.34 (8)	O23^vii^—Mn1—O22	159.42 (10)
O24^i^—K—O11^v^	114.87 (10)	O11—Mn1—O22	71.87 (10)
O24^ii^—K—O11^v^	99.26 (10)	O11^i^—Mn1—O22	90.70 (10)
O24^iii^—K—O11^v^	87.10 (9)	O22^i^—Mn1—O22	113.36 (14)
O11^iv^—K—O11^v^	52.22 (11)	O24^ii^—Fe2—O12^x^	94.65 (12)
O24—K—O22	53.32 (8)	O24^ii^—Fe2—O22	86.05 (12)
O24^i^—K—O22	121.80 (11)	O12^x^—Fe2—O22	109.44 (13)
O24^ii^—K—O22	57.49 (9)	O24^ii^—Fe2—O21^xi^	101.92 (12)
O24^iii^—K—O22	116.43 (11)	O12^x^—Fe2—O21^xi^	87.13 (12)
O11^iv^—K—O22	144.11 (8)	O22—Fe2—O21^xi^	161.16 (11)
O11^v^—K—O22	122.58 (8)	O24^ii^—Fe2—O11	101.11 (11)
O24—K—O22^i^	121.80 (11)	O12^x^—Fe2—O11	162.62 (11)
O24^i^—K—O22^i^	53.32 (8)	O22—Fe2—O11	79.18 (11)
O24^ii^—K—O22^i^	116.43 (11)	O21^xi^—Fe2—O11	82.52 (11)
O24^iii^—K—O22^i^	57.49 (9)	O24^ii^—Fe2—O21^vi^	174.59 (11)
O11^iv^—K—O22^i^	122.58 (8)	O12^x^—Fe2—O21^vi^	81.71 (11)
O11^v^—K—O22^i^	144.11 (8)	O22—Fe2—O21^vi^	91.37 (11)
O22—K—O22^i^	82.66 (13)	O21^xi^—Fe2—O21^vi^	81.97 (11)
O23^vi^—Mn3—O23^vii^	75.91 (14)	O11—Fe2—O21^vi^	83.04 (10)
O23^vi^—Mn3—O12^viii^	84.68 (13)	O12^xii^—P1—O12	104.0 (3)
O23^vii^—Mn3—O12^viii^	121.62 (16)	O12^xii^—P1—O11^xii^	107.33 (17)
Mn1^viii^—Mn3—O12	76.0 (3)	O12—P1—O11^xii^	114.23 (15)
O23^vi^—Mn3—O12	94.38 (14)	O12^xii^—P1—O11	114.23 (15)
O23^vii^—Mn3—O12	79.85 (14)	O12—P1—O11	107.33 (17)
O12^viii^—Mn3—O12	157.20 (11)	O11^xii^—P1—O11	109.7 (2)
Mn1^viii^—Mn3—O23^ix^	69.7 (3)	O24—P2—O23	110.71 (16)
O23^vi^—Mn3—O23^ix^	157.58 (11)	O24—P2—O22	109.32 (15)
O23^vii^—Mn3—O23^ix^	123.93 (15)	O23—P2—O22	111.67 (18)
O12^viii^—Mn3—O23^ix^	91.62 (14)	O24—P2—O21	110.22 (17)
O12—Mn3—O23^ix^	80.58 (13)	O23—P2—O21	108.50 (16)
O23^vi^—Mn1—O23^vii^	78.61 (15)	O22—P2—O21	106.32 (16)

Symmetry codes: (i) −*x*, *y*, −*z*+1/2; (ii) *x*, −*y*, *z*−1/2; (iii) −*x*, −*y*, −*z*+1; (iv) −*x*, −*y*, −*z*; (v) *x*, −*y*, *z*+1/2; (vi) −*x*+1/2, −*y*+1/2, −*z*+1; (vii) *x*−1/2, −*y*+1/2, *z*−1/2; (viii) −*x*, −*y*+1, −*z*; (ix) *x*−1/2, *y*+1/2, *z*−1; (x) *x*+1/2, −*y*+1/2, *z*+1/2; (xi) *x*, *y*, *z*−1; (xii) −*x*, *y*, −*z*−1/2.
